# Complete Genome Sequence of *Enterococcus faecalis* Strain P8-1 Isolated from Wild Magellanic Penguin (*Spheniscus magellanicus*) Feces on the South Coast of Brazil

**DOI:** 10.1128/genomeA.01531-15

**Published:** 2016-01-14

**Authors:** Janira Prichula, Fabricio Souza Campos, Rebeca Inhoque Pereira, Leonardo Almansa Cardoso, Guilherme Raffo Wachholz, Luiza Pieta, Roberta Fogliatto Mariot, Tiane Martin de Moura, Maurício Tavares, Pedro Alves d’Azevedo, Jeverson Frazzon, Ana Paula Guedes Frazzon

**Affiliations:** a Basic Health Sciences Institute, Federal University of Rio Grande do Sul (UFRGS), Porto Alegre, Brazil; b Department of Microbiology and Parasitology, Federal University of Health Sciences of Porto Alegre (UFCSPA), Porto Alegre, Brazil; c Food Science Institute, UFRGS, Porto Alegre, Brazil; d Center for Coastal Studies, Limnology and Marine, UFRGS, Imbé, Brazil

## Abstract

*Enterococcus faecalis* strains have a ubiquitous nature that allows them to survive in different niches. Studies involving enterococci isolated from marine animals are scarce. Therefore, in this study, we report the complete genome sequence of *E. faecalis* strain P8-1 isolated from feces of a Magellanic penguin on the south coast of Brazil.

## GENOME ANNOUNCEMENT

*Enterococcus* spp. are Gram-positive catalase-negative cocci, with an ability to tolerate broad ranges in pH and temperatures and variations in salinity, which allow them to survive in diverse ecological niches (1). *Enterococcus faecalis* is a commensal of the gastrointestinal tract of humans and animals and is widely distributed in soil, water, plants, and foods (2–4). However, it has been considered an important microbial with high clinical impact for its capacity to acquire and transfer resistance and virulence genes, which gives it selective advantages to survive and disperse in the environment (5, 6).

Studies involving enterococci isolated from wild marine animals are scarce, probably due to the migratory habits of some species and the difficulty in obtaining samples (7–9). In this direction, the work here presents the complete genome sequence of *E. faecalis* strain P8-1, which was isolated from feces of a wild Magellanic penguin. This strain contains multiple phage sequences and secondary metabolite clusters, such as microcin, lasso peptide, and bacteriocin, besides two confirmed clusters of regularly interspaced short palindromic repeat (CRISPR)-associated genes existing in the genome.

A library of the *E. faecalis* genomic DNA was prepared using the Nextera DNA library preparation kit with 24 samples (catalog no. FC-121-1030; Illumina, San Diego, CA), and the paired-end sequencing was performed on the Illumina MiSeq Platform using the MiSeq reagent kit version 3 with 150 cycles (catalog no. MS-102-3001; Illumina). The reads were subjected to *de novo* assembly using Andrew and Aaron’s Awesome Assembly pipeline (a5), and open reading frames (ORFs) were predicted using rapid prokaryotic genome annotation (Prokka). After assembly, a total of 42 contigs was generated for *E. faecalis* strain P8-1. Sequence assembly yielded 3,146,744 bp for the whole genome of *E. faecalis* strain P8-1, with a G+C content of 37.07%, and the longest scaffold size was of 421.685 bp, with an *N*_50_ of 194,850 bp and raw coverage of 140×.

### Nucleotide sequence accession number.

This whole-genome shotgun project has been deposited in GenBank under the accession number LKGR00000000. The version described in this paper is the first version.
